# Immunologic and Virologic Mechanisms for Partial Protection from Intravenous Challenge by an Integration-Defective SIV Vaccine †

**DOI:** 10.3390/v9060135

**Published:** 2017-06-02

**Authors:** Chu Wang, Chunlai Jiang, Nan Gao, Kaikai Zhang, Donglai Liu, Wei Wang, Zhe Cong, Chuan Qin, Vitaly V. Ganusov, Guido Ferrari, Celia LaBranche, David C. Montefiori, Wei Kong, Xianghui Yu, Feng Gao

**Affiliations:** 1 National Engineering Laboratory for AIDS Vaccine, School of Life Sciences, Jilin University, Changchun 130012, Jilin, China; wangchu13@mails.jlu.edu.cn (C.W.); jiangcl@jlu.edu.cn (C.J.); gaonan15@mails.jlu.edu.cn (N.G.); vocebianca@yeah.net (K.Z.); liudonglai@nifdc.org.cn (D.L.); weikong@mail.jlu.edu.cn (W.K.); 2 Key Laboratory for Molecular Enzymology and Engineering, the Ministry of Education, School of Life Sciences, Jilin University, Changchun 130012, Jilin, China; 3 Division II of In Vitro Diagnostics for Infectious Diseases, Institute for In Vitro Diagnostics Control, National Institutes for Food and Drug Control, Beijing 100050, China; 4 Institute of Laboratory Animal Science, Chinese Academy of Medical Sciences, Beijing 100021, China; wangw@cnilas.org (W.W.); congz@cnilas.org (Z.C.); qinchuan@pumc.edu.cn (C.Q.); 5 Comparative Medicine Center, Peking Union Medical College, Beijing 100021, China; 6 Department of Microbiology, University of Tennessee, Knoxville, TN 37996, USA; vitaly.ganusov@gmail.com; 7 Departments of Surgery, Duke University Medical Center, Durham, NC 27710, USA; gflmp@duke.edu (G.F.); celia.labranche@duke.edu (C.L.); david.montefiori@duke.edu (D.C.M.); 8 Departments of Medicine, Duke University Medical Center, Durham, NC 27710, USA

**Keywords:** SIV, vaccine, integration defection, challenge, single genome sequencing

## Abstract

The suppression of viral loads and identification of selection signatures in non-human primates after challenge are indicators for effective human immunodeficiency virus (HIV)/simian immunodeficiency virus (SIV) vaccines. To mimic the protective immunity elicited by attenuated SIV vaccines, we developed an integration-defective SIV (idSIV) vaccine by inactivating integrase, mutating sequence motifs critical for integration, and inserting the cytomegalovirus (CMV) promoter for more efficient expression in the SIVmac239 genome. Chinese rhesus macaques were immunized with idSIV DNA and idSIV particles, and the cellular and humoral immune responses were measured. After the intravenous SIVmac239 challenge, viral loads were monitored and selection signatures in viral genomes from vaccinated monkeys were identified by single genome sequencing. T cell responses, heterologous neutralization against tier-1 viruses, and antibody-dependent cellular cytotoxicity (ADCC) were detected in idSIV-vaccinated macaques post immunization. After challenge, the median peak viral load in the vaccine group was significantly lower than that in the control group. However, this initial viral control did not last as viral set-points were similar between vaccinated and control animals. Selection signatures were identified in Nef, Gag, and Env proteins in vaccinated and control macaques, but these signatures were different, suggesting selection pressure on viruses from vaccine-induced immunity in the vaccinated animals. Our results showed that the idSIV vaccine exerted some pressure on the virus population early during the infection but future modifications are needed in order to induce more potent immune responses.

## 1. Introduction

After over three decades of research, an efficient human immunodeficiency virus type 1 (HIV-1) vaccine is still elusive [1,2]. As successfully demonstrated for some infectious pathogens, live-attenuated vaccines have been effective ways to control infections that significantly affect public health. Similarly, attenuated simian immunodeficiency virus (SIV) vaccines induce effective protection from SIV challenge in non-human primates (NHPs) [3,4,5]. Attenuated SIV vaccines are usually engineered by inactivating non-essential regulatory genes to reduce viral replication capacity and pathogenicity. However, SIV mutants with multiple deleted accessary genes could still cause acquired immunodeficiency syndrome (AIDS) in infant and adult monkeys, and thus, similarly attenuated HIV-1 vaccines will not likely be used in humans [6]. Recent studies using a replication-competent cytomegalovirus (CMV) vector that can persistently express SIV genes showed a 50% protection efficacy in vaccinated macaques against the infection of a pathogenic autologous SIVmac239 challenge through non-classical CD8^+^ T immune responses [7,8]. The results from attenuated SIV vaccines and replication-competent CMV vector vaccines indicate that viral vector-based vaccines that can stimulate the immune system with all viral proteins without integration of the viral genome into host chromosomes may induce desirable protective immune responses.

During the life cycle of retrovirus replication, pre-integration complexes are imported into the nucleus of infected cells and some proviral genomes are integrated in the chromosome to complete virus replication. However, the majority of proviral genomes are present in extrachromosomal forms (linear and single or two long terminal repeat (LTR) circles), which can persist for about a month and can actively express viral proteins [9,10,11]. Based on this unique process, integrase (IN)-defective lentiviral vectors (IDLV) have been used for the development of safe vaccines [12,13,14,15,16]. After immunization with IDLV-based vaccines, persistent immune responses to the HIV-1 Env protein or long-term sterile protection against malaria were elicited [14,16,17,18]. These results suggest that persistent expression of several HIV/SIV proteins can lead to better immune responses needed for effective AIDS vaccines. One other important advantage of IDLV is that it eliminates the risk of insertional mutagenesis that can lead to cancer [19,20]. However, whether long lasting immune responses elicited by IDLV vaccines can protect against HIV/SIV infection has not been evaluated. 

To develop a vaccine that mimics the protective immunity elicited by attenuated SIV vaccines and takes advantage of the safety features of IDLV, we generated a new integration-defective SIV (idSIV) by expressing all viral proteins but without the integration of the SIV genome into host cell chromosomes. This novel idSIV vaccine contains the inactivated IN, deleted sequence motifs required for integration, and a CMV promoter to drive the expression of all viral proteins. We found that this idSIV vaccine significantly reduced the peak viremia after a vigorous intravenous challenge in NHPs but did not significantly reduce the viral set-point viral loads. By analyzing sequences from peak viremia, we identified six selection signatures among vaccinated macaques, which were absent in control animals, suggesting a selection pressure on challenge viruses. 

## 2. Results

### 2.1. Generation and Characterization of a Novel Integration-Defective SIV Vaccine

**Figure 1:**
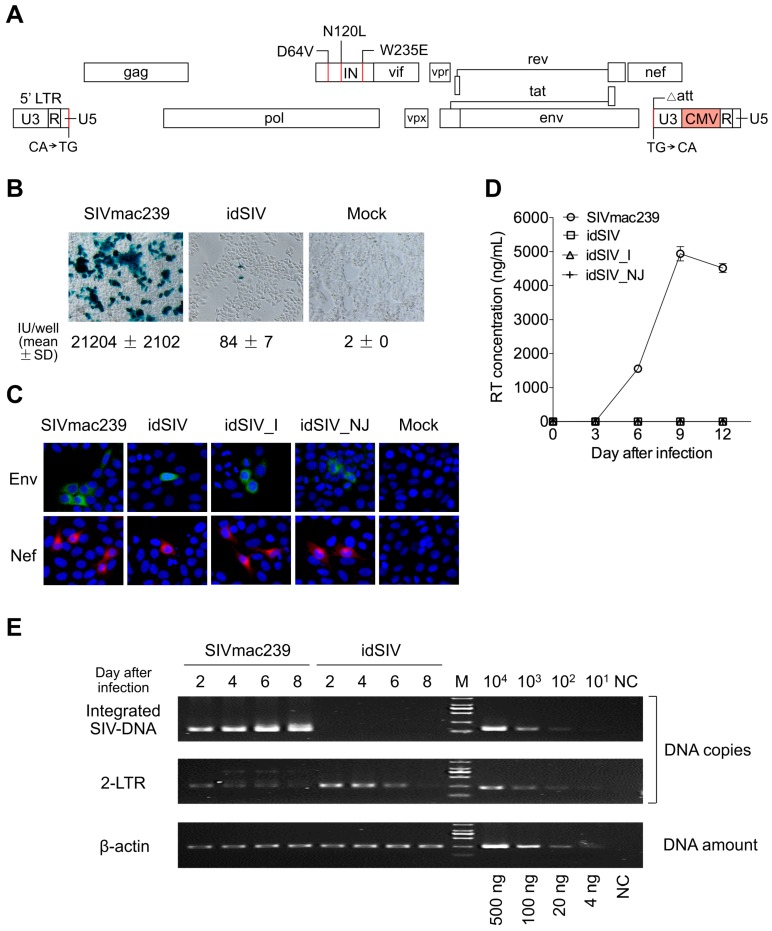
Construction and characterization of integration-defective SIV (idSIV). (**A**) Modifications of the infectious SIVmac239 clone. Three mutations (D64V, N120L, and W235E) were introduced in the integrase (IN) gene to abolish integration and replication activity. Highly conserved dinucleotides (CA) required for integration at both ends of long terminal repeats (LTRs) were mutated (TG→CA at the 3′-end and CA→TG at the 5′-end). The attachment site (*att*) critical for integration was deleted in 3′-LTR. In addition, a cytomegalovirus (CMV) promoter was inserted after the promoter region in U3 of the 3′-LTR. After reverse transcription, all modifications in 3′-LTR will be placed in both LTRs, and the CA→TG mutation will be transferred to the very end of 3′-LTR of the proviral genome (See Figure S1); (**B**) The infectivity of idSIV in TZM-bl cells. An equal amount (2.5 ng reverse transcriptase (RT)) of SIVmac239 or idSIV harvested from transfected 293T cells was used to infect TZM-bl cells. The infected cells were stained with 5-bromo-4-chloro-3-indolyl-β-d-galactopyranoside (X-Gal) and each blue cell was defined as an infectious unit (IU). The infected cells were counted under an optical microscope. All experiments were performed in triplicate; (**C**) Detection of the Env and Nef expression after idSIV infection. TZM-bl cells were infected with an equal amount (2.5 ng RT) of SIVmac239, idSIV, idSIV_I (pseudotyped with the Indiana serotype VSV-G), or idSIV_NJ (pseudotyped with the New Jersey serotype VSV-G). Expression of Env (green) and Nef (red) were detected with anti-Env and anti-Nef mAbs, respectively. Cellular nuclei were stained by 4′,6-diamidino-2-phenylindole (DAPI) (blue). Uninfected cells served as mock controls. The images were acquired using confocal laser scanning microscopy; (**D**) Viral replication in CEMx174 cells. An equal amount (0.45 ng RT) of SIVmac239, idSIV, idSIV_I, or idSIV_NJ harvested from the transfection of 293T cells was used to infect CEMx174 cells. Supernatants were collected every three days to measure RT concentrations. All experiments were performed in triplicate; (**E**) Detection of the integrated and unintegrated 2-LTR circle proviral DNA. Total cellular DNA was extracted from infected CEMx174 cells. The integrated DNA was detected by *Alu*-PCR, while the unintegrated 2-LTR circles were detected by specific primers that were in opposite orientations in 3′-U5 and 5′-U3. The DNA quality and integrity in all samples were determined by PCR amplification of cellular β-actin DNA. SD: standard deviation.

To generate a safe vaccine that can elicit protective immune responses with pan-viral proteins as in attenuated SIV vaccines, we constructed an idSIV by introducing a number of modifications into the SIVmac239 genome to avoid the risks due to integration and by enhancing the expression of viral proteins under a potent CMV promoter (Figure 1A). First, we introduced three class I mutations (D64V, N120L, and W235E) that can inactivate IN but avoid the reduction of IN expression to increase the level of unintegrated circular proviral DNA [21,22]. Second, dinucleotides CA that are required for integration at both ends of LTRs and the attachment site (*att*) for IN binding in the U3 region in the 3′-LTR were mutated and deleted, respectively, to ensure that no potential integration will be facilitated by residual integrase activity [22]. Third, the promoter in LTR was replaced with the CMV promoter by inserting it in the U3 region of the 3′-LTR so that the expression of all viral proteins was independent of regulation by the Tat protein. As a result, the proviral genome would not be integrated into host chromosomes and the viral protein expression was controlled by the potent CMV promoter (Figure S1). 

Western blot analysis of viral proteins in the cell lysates and purified virions from 293T cells transfected with idSIV or wild type (wt) SIVmac239 showed similar patterns (Figure S2). idSIV infected TZM-bl cells, but its infectivity was ~250 fold lower than that of wt SIVmac239 (Figure 1B). Both early (Nef) and later (Env) viral proteins were detected in TZM-bl cells after idSIV infection (Figure 1C). However, no SIV-infected cells were detected after passaging newly harvested idSIV to fresh TZM-bl cells. Similar results were observed in CEMx174 cells (Figure 1D).

After being pseudotyped with the envelope glycoprotein (VSV-G) Indiana (I) or New Jersey (NJ) serotype of the vesicular stomatitis virus (Figure S3A), idSIV_I and idSIV_NJ were 37.8 and 21.1 fold more infectious than idSIV (*p* < 0.0001 and *p* = 0.001, respectively), but both were still 14.8 and 26.5 fold less infectious than SIVmac239 (*p* < 0.0001) in TZM-bl cells, respectively (Figure S3B). VSV-G pseudotype viruses did not render idSIV infectious in CEMx174 cells over time (Figure 1D). Taken together, these results demonstrated that only a small portion of idSIV particles was able to express viral proteins after infection and that no infectious viruses were produced to establish a new infection. 

Integrated DNA was readily detected by *Alu*-PCR at day 2 post infection and continuously increased over time in SIVmac239 infected CEMx174 cells (Figure 1E and Figure S4). However, no integrated DNA was detected in idSIV-infected cells throughout the culture (Figure 1E and Figure S4A). The 2-LTR circle form of unintegrated extrachromosomal DNA (E-DNA) was 9.5 fold higher in idSIV infected cells than in wt SIVmac239 infected cells at day 2 (*p* = 0.0003), similar to previous reports with IN-defective retroviral vectors [11] (Figure 1E and Figure S4B). These results demonstrated that idSIV proviral DNA was not integrated into host chromosomes but existed as the E-DNA form after infection.

### 2.2. Elicitation of Cellular Responses

**Figure 2:**
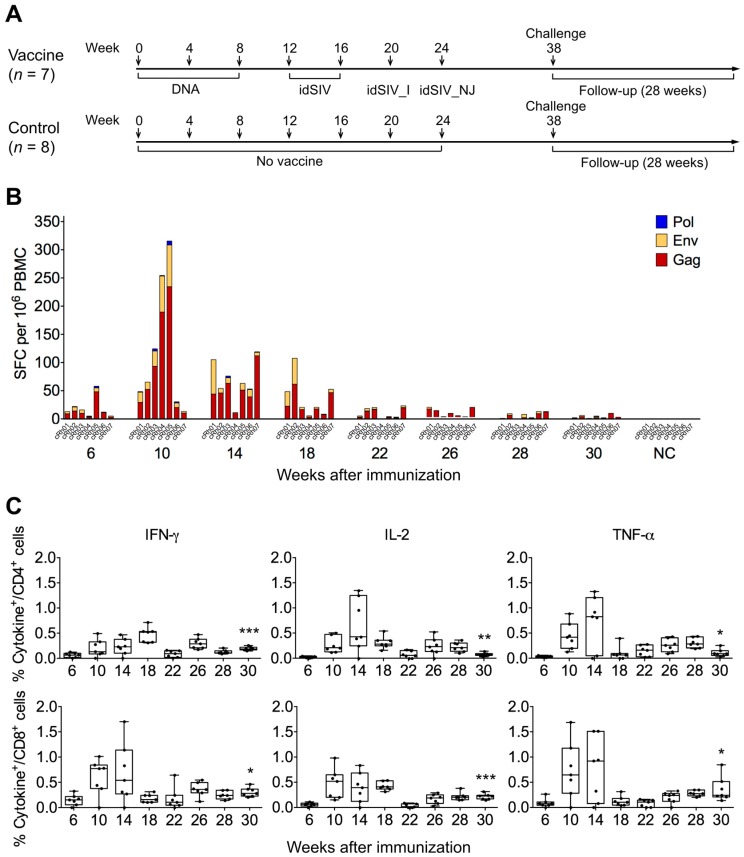
T cell responses elicited by idSIV immunization in monkeys. (**A**) Monkeys in the vaccine group (*n* = 7) received three idSIV DNAs (weeks 0, 4, and 8) and were then sequentially boosted with idSIV particles two times (weeks 12 and 16), one idSIV_I (week 20), and one idSIV_NJ (week 24). Eight control monkeys received phosphate buffered saline (PBS) each time; (**B**) Detection of T cell responses by enzyme-linked immunospot assay (ELISpot). T cell responses were determined with peripheral blood mononuclear cells (PBMCs) collected 2 weeks post each immunization as well as 10 and 8 weeks before challenge using the pooled Gag, Env, or Nef peptides from SIVmac239. Each column represents the combined spot forming cells (SFCs) with all three gene peptide pools from each monkey. A negative control (NC) was performed with cells collected from each monkey before immunization; (**C**) CD4^+^ and CD8^+^ T cell responses. The same PBMCs were stimulated with pooled SIVmac239 peptides and the frequencies of the IFN-γ, interleukin-2 (IL-2), and tumor necrosis factor-α (THF-α) producing T cells were determined by intracellular cytokine staining (ICS). The frequencies of CD4^+^ and CD8^+^ T cell responses between week 6 and week 30 were compared using unpaired Student’s *t*-test (* *p* < 0.05, ** *p* < 0.01, *** *p* < 0.001).

To determine if idSIV could induce T cell immune responses, we immunized seven Chinese rhesus macaques (cRh01 to cRh07) with three idSIV DNA doses (weeks 0, 4, and 8) and sequentially boosted them with idSIV particles two times (weeks 12 and 16), one idSIV_I (week 20), and one idSIV_NJ (week 24) (Figure 2A). Eight control monkeys (cRh08 to cRh15) received only phosphate buffered saline (PBS) each time. T cell responses were determined by peripheral blood mononuclear cells (PBMCs) collected two weeks after each immunization after the second DNA immunization (weeks 6, 10, 14, 18, 22, and 26) as well as 10 and 8 weeks before challenge (week 28 and 30). After PBMCs were stimulated with SIVmac239 Gag, Env, or Pol peptide pools, the number of spot forming cells (SFCs) secreting interferon-γ (IFN-γ) was determined by enzyme-linked immunospot assay (ELISpot). We focused on these three largest proteins encoded by SIV due to the availability of the SIVmac239 peptide sets and the limited volume of blood samples obtained from each macaque. T cell responses were detected in all monkeys after three DNA immunizations (week 10) although the levels were variable (Figure 2B). After two idSIV particle immunizations, the T cell responses decreased but were still maintained at relatively high levels (weeks 14 and 18). T cell responses were not boosted by idSIV_I and idSIV_NJ immunizations and were reduced to low levels by week 30. T cell responses were detected predominantly for Gag, at a reduced level for Env, and little (if any) for Pol (Figure 2B). We next performed the intracellular cytokine staining (ICS) assay to determine the CD4^+^ and CD8^+^ T cell responses by detecting IFN-γ, tumor necrosis factor-α (TNF-α), and interleukin-2 (IL-2). The dynamics of T cell responses determined by ICS and ELISpot was generally similar (Figure 2C). However, the potency of the T cell responses determined by ICS with any cytokine for CD4^+^ or CD8^+^ cells at week 30 were still maintained at higher levels than that after the second idSIV DNA immunization (*p* < 0.012). 

### 2.3. Humoral Responses in idSIV Vaccinated Macaques

**Figure 3:**
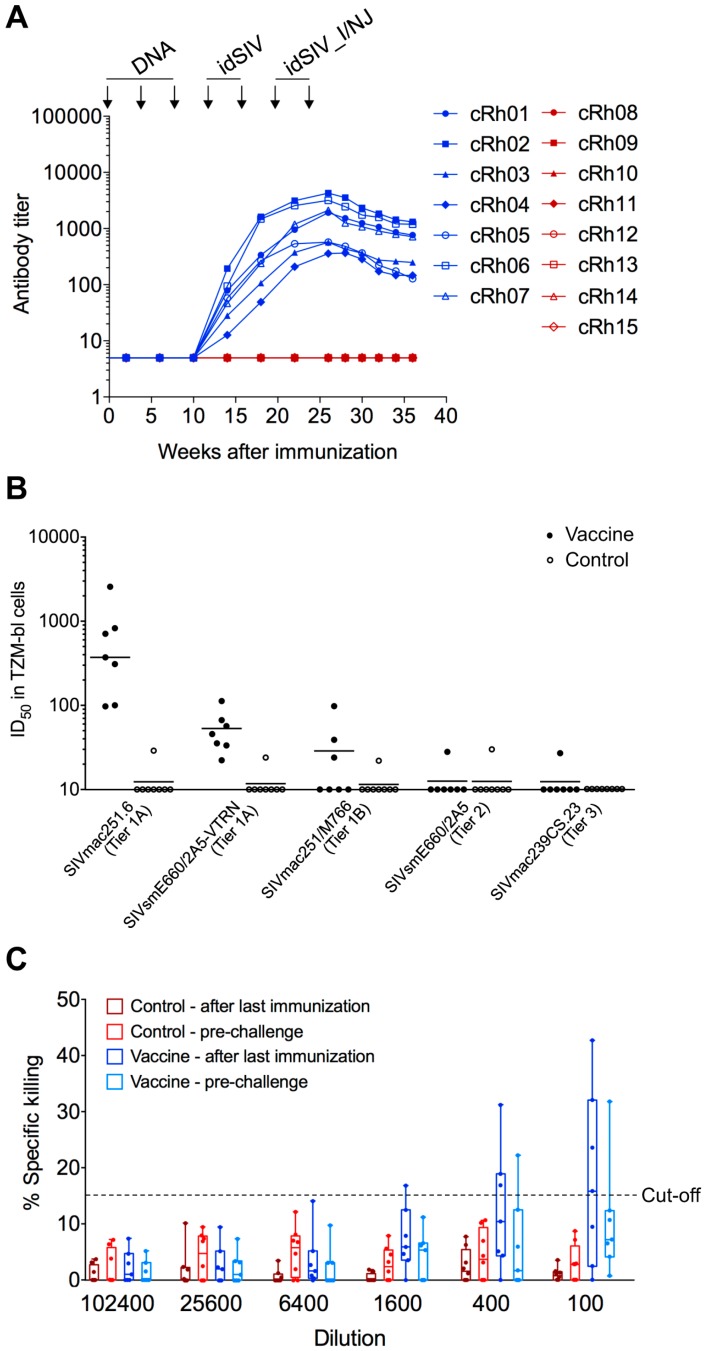
Humoral immune responses elicited by idSIV in monkeys. (**A**) Binding antibody titers to autologous SIVmac239 gp140 were measured by enzyme linked immunosorbent assay (ELISA). The time of immunizations are indicated by arrows; (**B**) Detection of neutralization activity. The 50% inhibition concentrations (ID_50_) are shown as the reciprocal titers of week 26 plasma samples (two weeks after the last immunization) against autologous SIVmac239 (tier-3) and heterologous tier-1 and tier-2 SIVs; (**C**) Detection of antibody-dependent cell-mediated cytotoxicity (ADCC) activity. ADCC activity in week 26 and 36 plasma samples was determined by a luciferase-based cytotoxicity assay. It was expressed as the percentage (%) of lysis of infected cells minus the background detected with the pre-immunization samples. Values above a threshold of 15% were considered positive.

Antibody responses to autologous SIVmac239 gp140 became detectable after the first idSIV virus particle immunization and were continuously boosted after each subsequent idSIV particle immunization (Figure 3A). The antibody (Ab) titers gradually decreased after the last immunization but were maintained at relatively high levels (<0.5 log decrease from the peak titer). Plasmas from all seven vaccinated monkeys could neutralize a tier-1A (more easy-to-neutralize) virus (SIVmac251.6) at higher dilutions and weakly neutralized another tier-1A virus (SIVsmE660/2A5-VTRN) (Figure 3B and Figure S5). Plasmas from three vaccinated macaques neutralized a tier-1B (less easy-to-neutralize) virus (SIVmac251/M766). Only the cRh01 plasma was able to neutralize a tier-2 (hard-to-neutralize) virus (SIVsmE660/2A5) and the autologous tier-3 (harder-to-neutralize) virus (SIVmac239). Some low levels of neutralizing antibody (nAb) titers (≤1:30) were detected in cRh08 and cRh09 in the control group, but the sera from both macaques did not neutralize SIVmac239 (Figure 3B and Figure S5). Neutralizing activity was not detected in week 36 samples from vaccinated macaques.

We next determined the ability of the same sera to mediate antibody-dependent cell-mediated cytotoxicity (ADCC) activity using target cells that were infected with SIVmac239. ADCC activity was detected in plasmas from four vaccinated macaques (cRh02, cRh03, cRh06, and cRh07) at a 1:100 dilution at week 26 (Figure 3C). At week 36, ADCC activity was only detectable in cRh02 (data not shown). No ADCC activity was detected in any macaques in the control group (Figure 3C). We also tested target cells pulsed with SIVmac239 gp120, but no ADCC activities were detected. These results suggested that the detected ADCC activities likely targeted epitopes naturally present on Env spikes expressed on the cell surface after infection. 

### 2.4. Reduced Peak Viremia in Vaccinated Monkeys after Challenge

**Figure 4:**
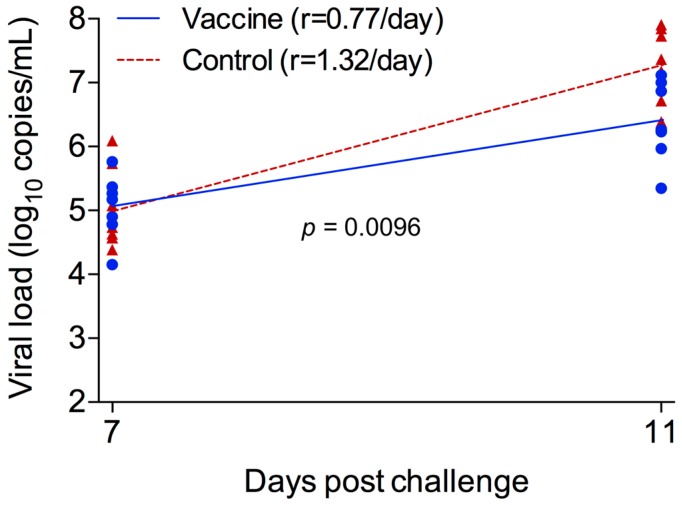
The rate of viral load increase during the ramp-up phase after challenge. The rates of viral load increase during day 7 to day 11 after challenge were compared between the vaccine and control groups using the bootstrap approach.

**Figure 5:**
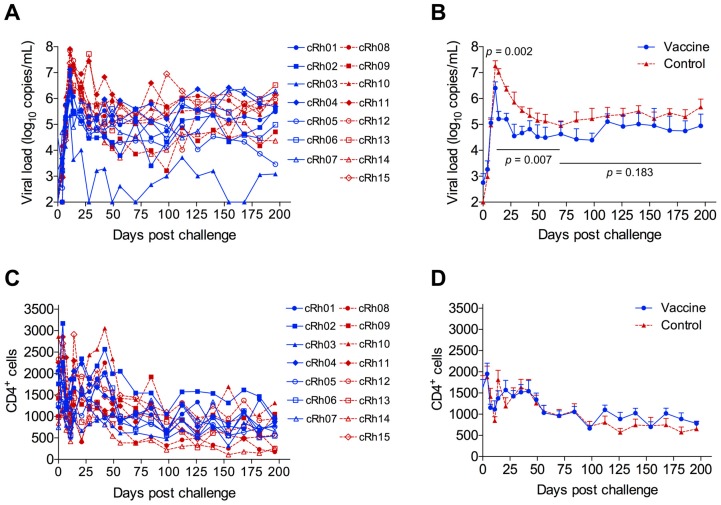
Dynamic changes of viral loads and CD4^+^ cells after challenge. (**A**) Viral loads in each individual monkey were monitored by quantitative real-time PCR (qRT-PCR) for 28 weeks after being challenged with wt SIVmac239; (**B**) Viral loads in vaccine and control groups were shown as mean + standard error of the mean (SEM). The differences of viral loads at peak viremia, the declining stage (day 11–70), or during the chronic phase (day 70–200) were compared between the vaccine group and the control group. CD4^+^ cell counts in each monkey and their mean + SEM in the vaccine; (**C**) and control (**D**) groups were measured during the 28-week follow-up period.

All monkeys were intravenously challenged with wt SIVmac239 (50% tissue culture infective dose (TCID_50_) = 50) 14 weeks after the last immunization (Figure 2A). During the virus load (VL) ramp-up stage (days 7–11), the accumulation rate of viral genome numbers was significantly lower in the vaccine group (0.77/day) than in the control group (1.32/day; *p* = 0.0096) (Figure 4). This led to a significantly lower mean peak viremia in the vaccine group (log_10_ 5.32 RNA copies/mL) than in the control group (log_10_ 6.62 RNA copies/mL; *p* = 0.002) (Figure 5A,B). The difference in VLs was as high as log_10_ 1.79 at day 14. During the VL declining stage (days 11–70), the VLs in the vaccinated animals were also significantly lower than those in the control group (*p* = 0.007). At the later set point, the VLs were similar between vaccinated and unvaccinated animals (*p* = 0.18). There were no differences in CD4^+^ T cell counts between the vaccine and control groups at any stages during infection (Figure 5C,D; *p* > 0.05).

### 2.5. Identification of Selection Signatures in Breakthrough Viruses

**Figure 6:**
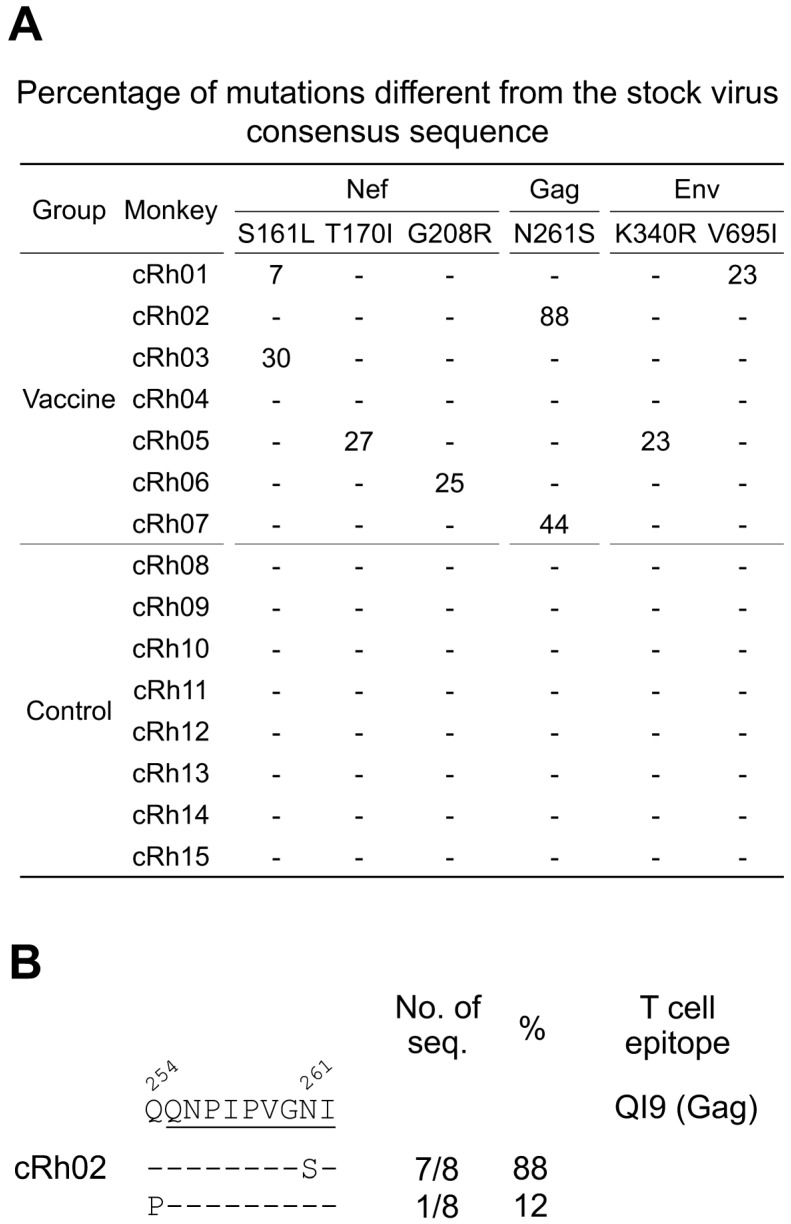
Genetic selection signature mutations in viral genomes. (**A**) Percentage of genetic selection signature mutations. The 3′-half genome and the *gag* gene sequences were obtained by single genome sequencing (SGS) for the SIVmac239 challenge stock and the plasma samples collected from each monkey at day 14 after infection. The sequence population from each monkey was compared to the consensus sequences of the SIVmac239 stock virus. The numbers are the percentages of mutations detected in viral populations. Only mutations that accounted for at least 20% in at least one monkey are shown; (**B**) A selection signature flanking the known T cell epitope QI9 was identified. Dashes indicate amino acids identical to the wt T cell epitopes.

Since the peak viremia was significantly reduced in the vaccinated monkeys, we sought to investigate if genetic selection signatures could be detected among vaccine breakthrough viruses. We first analyzed the viral population in the SIVmac239 challenge stock by characterizing the 3′-half genome and the *gag* gene sequences obtained by single genome sequencing (SGS) and found that the viral populations in both genomic regions were relatively homogenous (Figures S6–S8). Comparing the viral sequences obtained at day 14 after challenge to the stock consensus sequences, we identified six unique non-synonymous mutations that accounted for at least 20% of the viral population in each vaccine breakthrough animal. These mutations were detected in 20–40% of the viral populations, except for the N261S mutation that was detected in 88% of the viral population in cRh02 (Figure 6A). These six selection signatures were identified only in the vaccinated macaques (Figure 6A). Importantly, none of those sites were found in the challenge stock virus population (Figures S6 and S7). 

Three mutations (S161L, T170I, and G208R) were found in Nef. The S161L mutation, a known escape mutation in the Mamu-A*02-restricted epitope YY9 [23,24], was detected in cRh03 (Figure 6A). The T170I and G208R mutations were identified in cRh05 and cRh06, respectively. Another known escape mutation N261S in a well-studied Gag QI9 epitope restricted by Mamu-A*01 was detected in cRh02 and cRh07 (Figure 6A). The Q254P mutation flanking the QI9 epitope was also found in viral genomes which did not have the N261S mutation in cRh02 (Figure 6B). When both mutations were considered in the QI9 epitope, 100% of the viruses in cRh02 contained mutations in this region. Two mutations were found in Env regions in cRh01 and cRh05 (Figure 6A). The K340R mutation was in the V3 region, while the V695I mutation was found in the cytoplasm domain. Among those mutations, N261S and S161L were found in macaques carrying restriction Mamu-A*01 and Mamu-A*02 alleles, respectively (Table S1), suggesting that each of them could be selected by Mamu-A*01 or Mamu-A*02-restricted T cell responses. The N261S mutation in epitope QI9 was also found in cRh07. Since cRh07 did not have the Mamu-A*01 restriction allele, the mechanisms of accumulation of the N261S mutation were unclear. 

Six additional selection signatures were found in control animals. Three of them were only detected in the control animals, while three others were found in both the control and vaccinated animals (Figures S6 and S7). Since these mutations were detected only 14 days after challenge, they were not likely to be selected by T cell immune responses elicited by SIV after infection, because escape mutations were only detectable in <1% of the viral population after two weeks of infection [24,25]. Moreover, none of these mutations were found in known T cell epitopes. Importantly, minority populations were found in the challenge virus stock for four of those six mutations (23% in Pol, 15% in Vif, 8% in Env, and 8% in Nef). Thus, these mutations were most likely the results of the transmission of variants in the challenge stock and expansion in monkeys due to transmission fitness [26].

## 3. Discussion

We developed a new idSIV vaccine which expressed pan-viral proteins, elicited cellular and humoral immune responses, and reduced the peak viremia after the vigorous intravenous SIVmac239 challenge. However, this early control did not translate into a lower set point. Additional analysis showed no strong correlation between the magnitude of the immune response and peak viral load, possibly due to the relatively weak T cell and antibody responses (data not shown). Identification of selection signatures among breakthrough viruses in vaccinated macaques suggests that the immune responses induced by idSIV may have been strong enough to select escape mutations during acute infection. However, the strongest evidence for the vaccine efficacy is in the lower virus growth rate early in infection and lower peak viremia in vaccinated animals. 

The analysis of vaccine breakthrough virus sequences can identify immune selection signatures [27]. However, analysis of the *env* sequences in breakthrough NHPs has not identified signatures that are selected by vaccines after repeated low-dose mucosal challenges [28,29], even in the presence of potent autologous nAbs [30]. A 2-amino acid signature (AK) in the gp120 C1 region was reportedly enriched among the vaccine breakthrough viruses rather than among viruses in unvaccinated monkeys [31]. However, a later study showed that this signature was more likely a mucosal transmission signature and was not enhanced by immunization [26]. The lack of identified signatures selected by vaccine-elicited immune responses in breakthrough NHPs may be attributed to the highly heterologous virus population in the stock and low challenge doses that generally resulted in infection by a single transmission/founder (T/F) virus [26,28,29,30,31]. Although the viruses in the SIVmac239 challenge stock were relatively homogenous in this study, the limited mutations in each viral genome indicated that the viruses in the stock evolved into slightly different variants during short expansion in cell culture. Thus, upon transmission some mutations may be selected due to vaccine-elicited immune responses, increased transmission fitness, or simply by chance (founder effect). By comparing sequences from vaccinated animals to the stock virus consensus sequences, we identified six selection signatures in three viral proteins (Gag, Nef, and Env). Two of the selection signatures (S161L and N261S) are intriguing because they were found in the T cell epitopes previously described and both had been confirmed in previous studies as T cell escape mutations in Gag and Nef, respectively [23,24,32]. None of those mutations were detected among 52 sequences from the challenging stock. However, at present we do not have direct evidence that these mutations were driven by virus specific T cell responses.

There are several potential explanations for the detection of these selection signatures. First, the challenge stock may contain these specific signature mutations at low frequencies at which they were not detected by a small number of analyzed sequences. Second, these mutations may appear early during virus dynamics either by chance or because they improve virus replication. Third, and finally, these mutations could be selected by vaccine-induced immune responses. It was not possible to discriminate between these alternative mechanisms, but the different selection pressures between the vaccine and control groups points to escape from immune responses as a potential mechanism. All selection signature mutations in the vaccine group were not detected in the stock, while these mutations in the control group were mostly (four out of six) derived from those present at high frequencies (8–23%) in the stock. This suggests that the immune responses elicited by idSIV might have a certain selection pressure on the majority of viruses and may have selected rare mutations that were not detected by the limited sequences. On the other hand, without pre-existing immune responses in the control macaques, viruses with higher frequency mutations in the stock are more likely to be transmitted in the control macaques, possibly due to better transmission fitness [26]. 

We could not directly test the hypothesis that selection signatures arose due to T cell escape because all viable cells have been exhausted in this study; our conjecture that these signatures are due to escape from T cell immunity remains to be tested. Future studies are planned to test if those mutations are indeed targeted by the idSIV vaccine. The potential protection could not be due to the presence protective alleles in vaccine groups since both groups had similar monkeys with known protective alleles (one with Mamu-A*01 and one with Mamu-A*02 in the vaccine group; three with Mamu-A*02 in the control group, Table S1). The detection of low percentages of selection signatures right after the peak viremia indicated that the immune responses elicited by idSIV could quickly select escape mutations. However, the immune responses were not strong enough to suppress VLs at lower levels at set points. It will be important to elicit immune responses that can potently suppress virus replication or select mutations with significant fitness loss which cannot be easily restored by compensatory mutations [33,34,35,36,37,38]. 

The advantage of the idSIV vaccine is that it expresses all viral proteins after immunization and does not express any non-viral proteins. Thus, it can be repeatedly used without concerns of induction of anti-vector immune responses. The low and transient infectivity of idSIV might allow small amounts of viruses to be produced and stimulate the immune system after idSIV immunization. Safety concerns are always paramount for the use of non-integrated SIV vaccines. Importantly, idSIV has more safety features than previous constructs (inactivated IN, enhanced circular E-DNA, mutated dinucleotide CA at both ends of the LTRs, and the deleted *att* domain). No integrated proviral genomes were detected by the highly sensitive *Alu*-PCR, as previously reported for other non-integrated viral vectors [10,12,13,14,15,18]. More importantly, no continuous viral replication was detected in the cell culture. Furthermore, we obtained 272 3′-half genome sequences from seven immunized macaques. No sequences were found to contain any modified sequences in the idSIV genome. This further indicated no detectable integrated idSIV genomes in vaccinated macaques. Our results demonstrated that idSIV is safe without detectable integration in vitro. However, more rigorous in vivo studies are needed to further confirm the lack of persistence of integrated DNA after idSIV immunization. Overall, our results suggest that the idSIV vaccine shows some promise by providing protection early after challenge, but further modifications to the vaccine and/or vaccination schedule are needed before moving this vaccine forward. 

## 4. Materials and Methods

### 4.1. Generation of the Full-Length idSIV Genome

To generate the idSIV molecular clone, we synthesized complete proviral SIVmac239 genome in three fragments (GeneScript, Piscataway, NJ, USA) with the following modifications: three class I mutations (D64V, N120L, and W235E) which are known to inactivate IN, minimally affect IN expression, and produce more unintegrated circular proviral DNA; dinucleotides CA to TG mutations at both ends of LTRs; deletion of the *att* domain in the U3 region in the 3′-LTR; and a 588-bp CMV promoter fragment corresponding to bases 232 to 819 for pCMVPA1 was inserted between the end of the U3 promoter and the R region. The final idSIV genome was confirmed by sequencing. 

### 4.2. Generation of Purified idSIV Particles

The idSIV DNA was transfected into 293T cells using Lipofectamine 2000 (Invitrogen Corp., Carlsbad, CA, USA). Virus particles in the supernatant of transfected 293T cells were harvested 72 h after transfection. Culture supernatants were centrifuged at 3000× rpm for 10 min, filtered through a 0.22 μm membrane and concentrated through a 20% sucrose cushion by ultracentrifugation at 100,000× *g* for 2 h at 4 °C. The virus pellets were re-suspended in sterilized PBS and stored at −80 °C for further analyses. Concentrations of purified virion particles were determined by measuring the RT concentration using the Lenti RT Activity Kit (Cavidi, Uppsala, Sweden).

To increase the infectivity of idSIV, idSIV was pseudotyped with VSV-G (gifts from John K Rose). Pseudoviruses with the Indiana strain (idSIV_I) or the New Jersey strain (idSIV_NJ) were generated by cotransfecting idSIV and VSV-G plasmid DNA at a molar ratio of 2:1. 

### 4.3. Detection of Integrated and Unintegrated Proviral Genomes

CEMx174 cells (2 × 10^5^/well) were infected with DNase I (1 U/10 μL) treated SIVmac239 or idSIV in 6-well plates at 37 °C for 4 h. Same amounts of idSIV and SIVmac239 (3800 IUs) were used to infect CEMx174 cells. Because the infectivity of idSIV was 250-fold lower than that of SIVmac239, the virus input for idSIV was increased by 250 fold (112.5 ng and 0.45 ng RT for idSIV and SIVmac239, respectively). After three washes, cells were cultured for 8 days. Culture supernatants were harvested every two days and replenished with the fresh complete medium. Total cellular DNA was extracted using a DNeasy Blood and Tissue kit (Qiagen, Frederick, MD, USA). Detection of integrated SIV genomes was performed with 20 ng of total DNA using an *Alu*-PCR assay as previously described [10]. The cell quality and numbers used for *Alu*-PCR were equilibrated by measuring human β-action gene copies. PCR products were visualized and analyzed by gel electrophoresis on 2% agarose gels. Unintegrated 2-LTR SIV proviral genomes were analyzed with 2 ng of total DNA by nested PCR [39]. First-round PCR was performed with 0.2 µM of forward primer 2-LTR-F1 (5′-GAAGTAAGCTAGTGTGTGTTCCC-3′; nucleotide (nt) 10,150–10,172) and reverse primer 2-LTR-R1 (5′-TGTCTTTGGGTATCTAATTCCTG-3′; nt 103–125), and 0.5 U High Fidelity Platinum Taq DNA Polymerase (Invitrogen) in a 20 µL reaction containing High fidelity buffer, 2 mM MgSO_4_, and 0.2 mM of deoxy-ribonucleoside triphosphates (dNTPs). PCR conditions were 94 °C for 2 min, followed by 35 cycles of 94 °C for 15 s, 55 °C for 30 s, and 68 °C for 5 min, with a final extension period of 10 min at 68°C. Second-round PCR was performed with 0.2 µM of forward primer 2-LTR-F2 (5′-CTAGTGTGTGTTCCCATCTCTCC-3′; nt 10,158–10,180) and reverse primer 2-LTR-R2 (5′-TGTAATCCTGCCAATCTGGTATG-3′; nt 71–93), 1.25 U High Fidelity Platinum Taq DNA Polymerase and 2 µL of the first-round product in a 50 µL reaction. The PCR conditions were the same as for the first-round PCR, except that the annealing temperature was increased to 60 °C. A 2-LTR plasmid was constructed as a standard by inserting a complete 2-LTR region (cloned by amplification of infected cells with 5′-GCTCTAGAGAAGTAAGCTAGTGTGTGTTCCC-3′ and 5′-CGGGATCCTGTCTTTGGGTATCTAATTCCTG-3′) into pVR1012 at the Xba I and BamH I restriction sites.

### 4.4. Viral Infectivity Assay

The infectivity of idSIV was determined by a multinuclear activation of a galactosidase indicator assay. Briefly, TZM-bl cells were plated at 10^5^ per well in 24-well plates one day before infection. The cells were at 20 to 30% confluence on the day of infection and the medium was removed before incubation. Equal amounts of viruses (2.5 ng RT) in 10 uL were mixed with 20 mM diethylaminoethyl (DEAE)-dextran per well in 200 μL of DMEM, and the mixture was then incubated with TZM-bl cells for 1.5 h at 37 °C, followed by addition of 1 mL complete Dulbecco’s modified eagle medium (DMEM) (DMEM with 10% fetal bovine serum (FBS). After incubation for 48 h, 5-bromo-4-chloro-3-indolyl-β-d-galactopyranoside (X-Gal) was added to the TZM-bl cells. The viruses harvested from the infected TZM-bl cells were used to infect fresh TZM-bl cells. Each positive blue cell was counted as one infectious unit. All experiments were performed in triplicate. To determine if idSIV could continuously infect fresh target cells, same amounts of viruses (0.45 ng RT) from transfected 293T cells were incubated with 2 × 10^5^ CEMx174 cells at 37 °C for 4 h. After the cells were washed three times, cells were cultured for 12 days. Virus replication was monitored every three days by measuring RT concentrations in culture supernatants.

### 4.5. Immunization and Challenge of Rhesus Macaques

Chinese rhesus macaques were maintained in the Institute of Laboratory Animal Science, Chinese Academy of Medical Sciences, and Peking Union Medical College (an AAALAC accredited facility). All experiments were performed in accordance with the relevant guidelines and regulations. All experimental protocols were approved by the Institutional Animal Care and Use Committee (IACUC) of the Institute of Laboratory Animal Science (approval number: ILAS-VL-2014-003). Macaques were genotyped for the Mamu-A*01, Mamu-A*02, Mamu-B*08, and Mamu-B*17 alleles as described previously. Of the 15 macaques, only one was Mamu-A*01 positive (cRh02), and four (cRh03, cRh10, cRh11, and cRh12) were *Mamu*-A*02 positive (Table S1). None of the macaques had the Mamu-B*08 or *Mamu*-B*17 allele. Tripartite motif-containing protein 5 alpha (TRIM5α) was not screened since some studies showed that it did not affect SIV replication in vaccinated and unvaccinated monkeys [40,41]. In the vaccine group, seven monkeys (cRh01–cRh07) were first immunized with DNA (5 mg) three times at weeks 0, 4, and 8, two idSIV virion particles (150 ng RT) at weeks 12 and 16, one idSIV_I (100 ng RT) at week 20, and one idSIV_NJ (100 ng RT) at week 24. DNA was delivered intramuscularly while idSIV and VSV-G pseudotyped idSIV virion particles were given by intravenous injection. Eight control monkeys (cRh08–cRh15) received an equal volume of PBS each time. Blood samples were collected regularly, and PBMC and plasma were separated for assessment of T and B cell immune responses. Fourteen weeks after the final immunization, all animals were intravenously challenged with 50 TCID_50_ of SIVmac239 (a gift from Preston A. Marx).

### 4.6. Determination of T Cell Immune Response

The IFN-γ ELISpot assay was performed using a monkey IFN-γ T cell ELISpot kit (U-CyTech, Utrecht, The Netherlands). PBMCs (3 × 10^5^ cells/well) in 96-well plates were stimulated in triplicate with peptide pools derived from SIVmac239 Gag, Env, and Pol proteins (15-mers overlapping by 11 amino acids; NIH AIDS Reagent Program (NARP)). The final concentration of each individual peptide was 5 µg/mL. Fifty ng/mL phorbol myristate acetate (PMA) and 1 µg/mL ionomycin (Sigma Chemicals, Co., St. Louis, MO, USA) was used as a positive control. SFCs were counted by CTL-ImmunoSpot S5 Analyzer (Cellular Technology Ltd, Shaker Heights, OH, USA) and expressed as SFC/10^6^ cells. Background (mean of wells without peptide) levels were subtracted from each well on the plate. A response was considered positive if the mean number of SFCs of triplicate sample wells exceeded the background plus two standard deviations (SDs). 

ICS was performed by incubating 10^6^ PBMCs with the SIVmac239 peptide pool (2 μg/mL for each peptide), PMA (50 ng/mL), or medium only (mock stimulation) in 500 µL of complete Roswell Park Memorial Institute (RPMI) 1640 medium with 10% FBS at 37 °C with 5% CO_2_ for 16 h. Two hours after stimulation, 1 µg/mL of Brefeldin A (Biolegend, Co., San Diego, CA, USA) was added to the cultures to inhibit cytokine secretion. Cells were stained with direct fluorochrome conjugates APC-CyTM7 mouse anti-human CD8 (RPA-T8), PerCP-CyTM5.5 mouse anti-human CD4 (L200), or PE-CyTM7 mouse anti-human CD3 (SP34-2) (BD Biosciences, San Diego, CA, USA), washed, and fixed with an IC fixation buffer (eBioscience, San Diego, CA, USA), permeabilized in a permeabilization buffer (eBioscience), and stained with APC-labeled anti-human IFN-γ Mab (4S.B3), FITC-labeled anti-human TNF-α (Mab11), or PE-labeled anti-human IL-2 (MQ1-17H12), or their isotype-matched controls (Biolegend). Samples were washed and analyzed with FACS canto I (BD Biosciences).

### 4.7. SIV Specific Binding Antibody Assay

Anti-SIV Env binding antibodies in monkey plasma samples were determined using autologous SIVmac239 gp140 protein by enzyme linked immunosorbent assay (ELISA). Antibody titers were calculated as the reciprocal dilution at which the mean ∆ODs (defined as the difference between the mean optical density (OD) of a dilution of a sample tested in two antigen-coated wells and the mean OD of the same dilution of a sample tested in two antigen-free wells) vs. Ln dilution crossed the cut-off OD (0.2). 

### 4.8. Neutralization Assay

Neutralization activity was measured in a luciferase reporter system in TZM-bl cells as described previously [42]. Plasma samples were heat-inactivated at 56 °C for 1 h and were then diluted at a 1:3 serial dilution starting at 1:20. The diluted plasma samples in duplicates were incubated with pseudoviruses for 1 h at 37 °C and then used to infect TZM-bl cells. The 50% inhibitory dose (ID_50_) was defined as the plasma dilution at which relative luminescence units (RLU) were reduced by 50% compared to the RLU in virus control wells after subtraction of the background RLU in cell control wells. A response was considered positive for neutralization if the ID_50_ titer was >1:20 dilution.

### 4.9. ADCC Assays

We tested the samples using the Luciferase-based (Luc) ADCC assay against the SIVmac239 IMC-infected target cells by adapting a previous methodology [43,44]. This is an ecto-IMC generated using the HIV-1 NL4-3 backbone with the insertion of the SIVmac239 envelope and the Luciferase reporter genes (kindly provided by C. Ochsenbauer at University of Alabama at Birmingham). The analysis of the results was conducted after subtracting the background detected with the pre-immunization samples. After background subtraction, the results would be considered positive if the % specific killing is above 15%. The samples collected before the first immunization, two weeks after the last immunization and two weeks before the challenge were analyzed.

### 4.10. Plasma Viral Load Assay

Viral RNA was isolated from plasma and used for complementary DNA (cDNA) synthesis by reverse transcription. The copy numbers of cDNA were detected using a TaqMan real-time PCR technique (Gag91F: 5′-GCAGAGGAGGAA ATTACCCAGTAC-3′, nt 1441–1446 in SIVmac239 and Gag91R: 5′-CAATTTTACCCAGGCATTTAATGTT-3′, nt 1508–1532) and an ABI 7500 Real-Time PCR System (Applied Biosystems Inc., Carlsbad, CA, USA). The TaqMan probe (5′-TGTCCACCTGCCATTAAGCCCGA-3′, nt 1484–1506) was labeled at the 5′ end with the reporter dye FAM (6-carboxyfluorescein) and at the 3′ end with the quencher dye TAMRA (6-carboxytetram ethyl-rhodamine). Serial dilutions of in vitro transcripts of SIV *gag* were used to generate a standard curve for each run. The copy numbers were determined by automated interpolation onto the standard curve generated by the ABI 7500 software v2.0.5 (Applied Biosystems Inc.).

### 4.11. Viral Sequence Analysis

The 3′-half genome and full *gag* gene sequences from the challenge stock and viruses at peak viremia were obtained by SGS as previous described [45,46]. Selection signatures were determined by comparing the peak viremia virus sequences to the consensus sequence of the challenge stock virus. Mutations that differed from the stock viruses by at least 20% were considered as selection signatures. GenBank accession numbers for the sequences generated in this study are: 3′-half genome sequences: KY359620–KY360240; *gag* sequences: KY359408–KY359619.

### 4.12. Statistical Analysis

The values of viral loads were presented as means + standard error of the mean (SEM). To compare viral loads between vaccine and control groups, we calculated the average viral load per time interval per animal and then compared these averages for vaccinated and unvaccinated animals using non-parameteric (Mann-Whitney) test. Intervals were 0–11 days (virus expansion phase), 11–70 days (virus contraction phase), and 70–270 days (set-point). To calculate the rate of viral load increase during the ramp-up phase, we used linear regression for log-transformed viral load data, and to compare slopes, we used the bootstrap approach (by resampling the data and calculating the number of times when the difference between the slopes was negative). Other statistical analyses were performed using PRISM v6 (GraphPad Software, Inc., La Jolla, CA, USA).

## Supplementary Materials

The following are available online at www.mdpi.com/1999-4915/9/5/135/s1. Figure S1: Schematic presentation of genomic replication of idSIV after transfection and infection. Figure S2: Analysis of idSIV viral proteins after transfection. Figure S3: Enhancement of idSIV infectivity by VSV-G. Figure S4: Detection of integrated and unintegrated proviral genomes after idSIV infection. Figure S5: Detection of neutralizing activity. Figure S6: Highlighter plot analysis of 3′-half genome sequences. Figure S7: Highlighter plot analysis of the *gag* gene sequences. Figure S8: Phylogenetic tree analysis of sequences from the SIVmac239 challenge stock. Table S1: MHC typing of Chinese rhesus macaques.
