# Supplementary material for: Immunologic and Virologic Mechanisms for Partial Protection from Intravenous Challenge by an Integration-Defective SIV Vaccine
Source: Viruses. 2017 Jun 2;9(6):135. doi: 10.3390/v9060135 (PMC5490812; doi:10.3390/v9060135)
Supplement: Supplementary file 1 [file viruses-09-00135-s001.pdf]

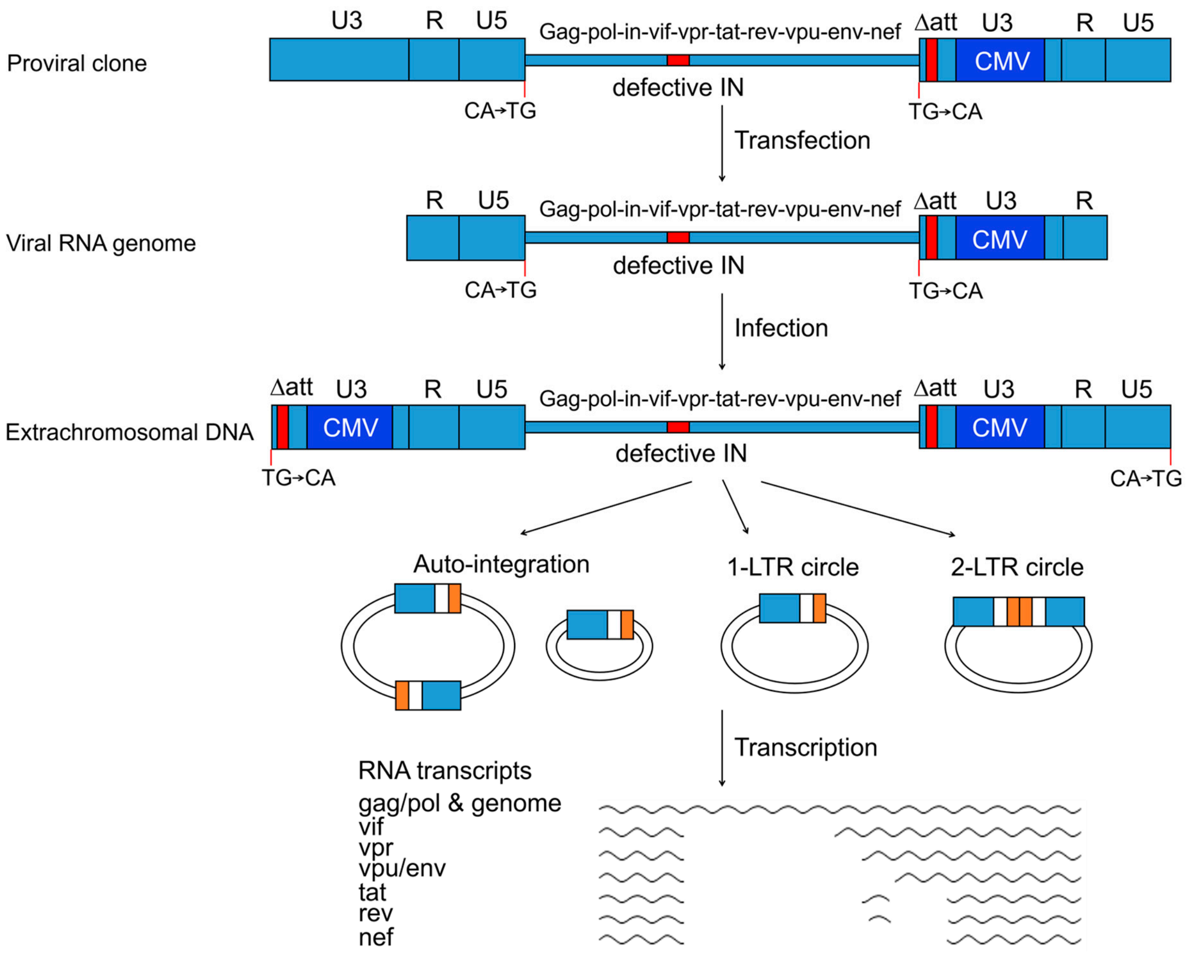

Figure S1. Schematic presentation of genomic replication of idSIV after transfection and infection. After transfection of idSIV plasmid DNA into 293T cells, the RNA genomes with all modifications are generated and packaged into virion particles. After infection of target cells by idSIV, the idSIV RNA genome is reverse transcribed into proviral genome, in which all modifications in the 3'-LTR (the addition of the CMV promoter, the att deletion, and the CA->TG mutation in the 3'-U3 region) are placed in the 5'-end LTR while the TG->CA mutation in the 5'-LTR is placed in the 3'-end LTR. Such proviral SIV genomes will be present as extrachromosomal forms and the transcription of viral genes is under control of the potent CMV promoter [1,2].

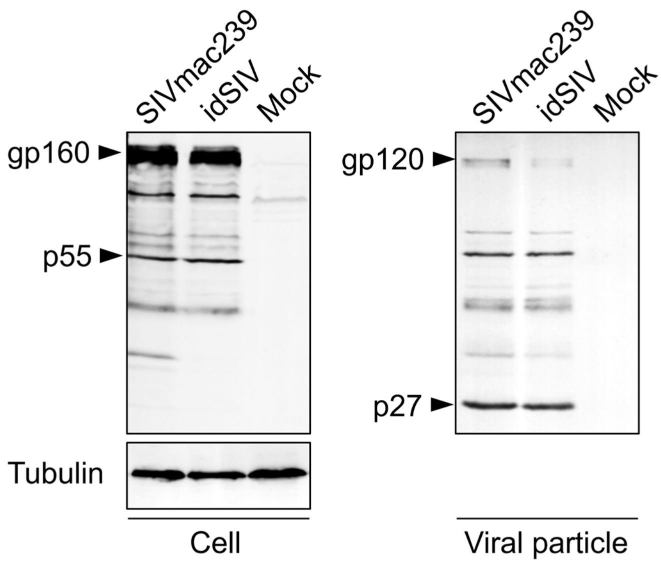

Figure S2. Analysis of idSIV viral proteins after transfection. 293T cells were transfected with wt SIVmac239 or idSIV DNA. Cell culture supernatants were harvested and transfected cells were lysed 48 hours after transfection. Viral proteins were analyzed by Western-blot using the SIVmac251-infected monkey serum. Cell lysates were equilibrated by host protein tubulin concentration. Non-transfected 293T cells and their supernatants were used as mock controls.

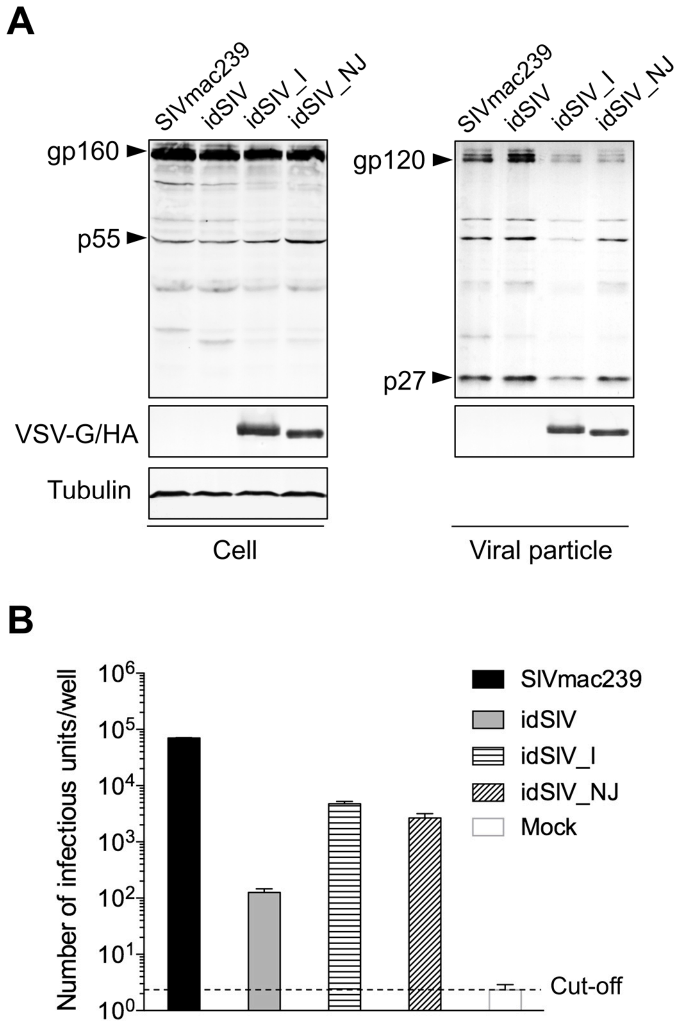

Figure S3. Enhancement of idSIV infectivity by VSV-G. (**A**) Protein expression of idSIV and VSV-G pseudotyped idSIVs after transfection. 293T cells were transfected with SIVmac239, idSIV, or idSIV, and VSV-G. Cell culture supernatants were harvested and transfected cells were lysed 48 hours after transfection. Viral proteins were analyzed by Western-blot using the SIVmac251-infected monkey serum. Cell lysates were equilibrated by tubulin concentration. The VSV-G/HA fusion proteins were detected with an anti-HA MAb. (**B**) Infectivity of idSIV and VSV-G pseudotyped idSIVs. TZM-bl cells were infected with equal amount (2.5 ng RT) of SIVmac239, idSIV, idSIV_I, or idSIV_NJ harvested from transfected 293T cells. Infected cells were counted in each well in a 24-well plate under an optical microscope. Each infected cell was counted as one infectious unit (IU). Uninfected cells served as mock controls. All experiments were performed in triplicate.

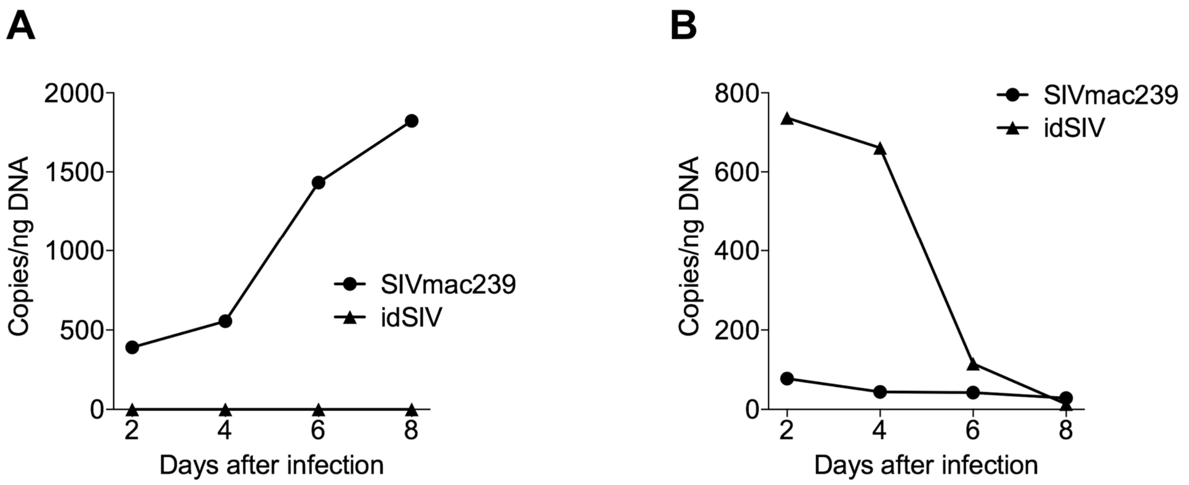

Figure S4. Detection of integrated and unintegrated proviral genomes after idSIV infection. Equal amounts of wt SIVmac239 or idSIV (3,800 IUs) were used to infect CEMx174 cells. Cells were harvested every two days post infection. Total DNA was isolated. (**A**) Integrated proviral DNA was amplified by Alu-PCR. (**B**) 2-LTR circles were amplified with PCR primers specific for 2-LTR circles. The PCR products were then subjected to qRT-PCR quantification using nested primers.

Group	Monkey	SIVmac251.6 (Tier 1A) Pre	SIVmac251.6 (Tier 1A) Post	SIVsmE660/2A5-VTRN (Tier 1A) Pre	SIVsmE660/2A5-VTRN (Tier 1A) Post	SIVmac251/M766 (Tier 1B) Pre	SIVmac251/M766 (Tier 1B) Post	SIVsmE660/2A5 (Tier 2) Pre	SIVsmE660/2A5 (Tier 2) Post	SIVmac239CS.23 (Tier 3) Pre	SIVmac239CS.23 (Tier 3) Post	SVA Pre	SVA Post
Vaccine	cRh01	<20	99	<20	66	30	72	<20	28	<20	27	<20	<20
	cRh02	<20	2541	<20	33	<20	39	<20	<20	<20	<20	<20	<20
	cRh03	<20	817	<20	22	30	29	<20	<20	<20	<20	<20	<20
	cRh04	<20	306	<20	35	41	35	<20	<20	<20	<20	<20	<20
	cRh05	24	96	<20	56	49	84	<20	<20	<20	<20	<20	<20
	cRh06	<20	368	<20	111	<20	98	<20	<20	<20	<20	<20	<20
	cRh07	<20	700	<20	45	<20	24	<20	<20	<20	<20	<20	<20
Control	cRh08	<20	29	23	61	30	68	<20	30	<20	<20	<20	<20
	cRh09	<20	<20	<20	24	<20	22	<20	<20	<20	<20	<20	<20
	cRh10	22	<20	39	<20	32	<20	25	<20	24	<20	<20	<20
	cRh11	<20	<20	<20	<20	<20	<20	<20	<20	<20	<20	<20	<20
	cRh12	<20	<20	20	<20	25	<20	<20	<20	<20	<20	<20	<20
	cRh13	<20	<20	25	<20	26	<20	<20	<20	<20	<20	<20	<20
	cRh14	<20	<20	<20	<20	<20	<20	<20	<20	<20	<20	<20	<20
	cRh15	<20	<20	<20	<20	<20	<20	<20	<20	<20	<20	<20	<20

Legend: > 1000 (red); 501-1000 (orange); 101-500 (yellow); 20-100 (green)

Figure S5. Detection of neutralization activity. The 50% inhibition concentrations (ID_{50}) are shown as the reciprocal titers of pre- and post-immunization plasma samples against autologous SIVmac239 (tier 3) and heterologous tier 1 and tier 2 SIVs. Pre: pre-immunization; Post: two weeks after the last immunization. Values are considered positive for any neutralization titers when neutralization activity was not detected in pre-immunization samples at a 1:20 dilution or for neutralization titers that are 3 times more than the titer detected for the pre-immunization samples from the same animal. The envelope glycoprotein (SVA) from the murine leukemia virus served as a non-specific control.

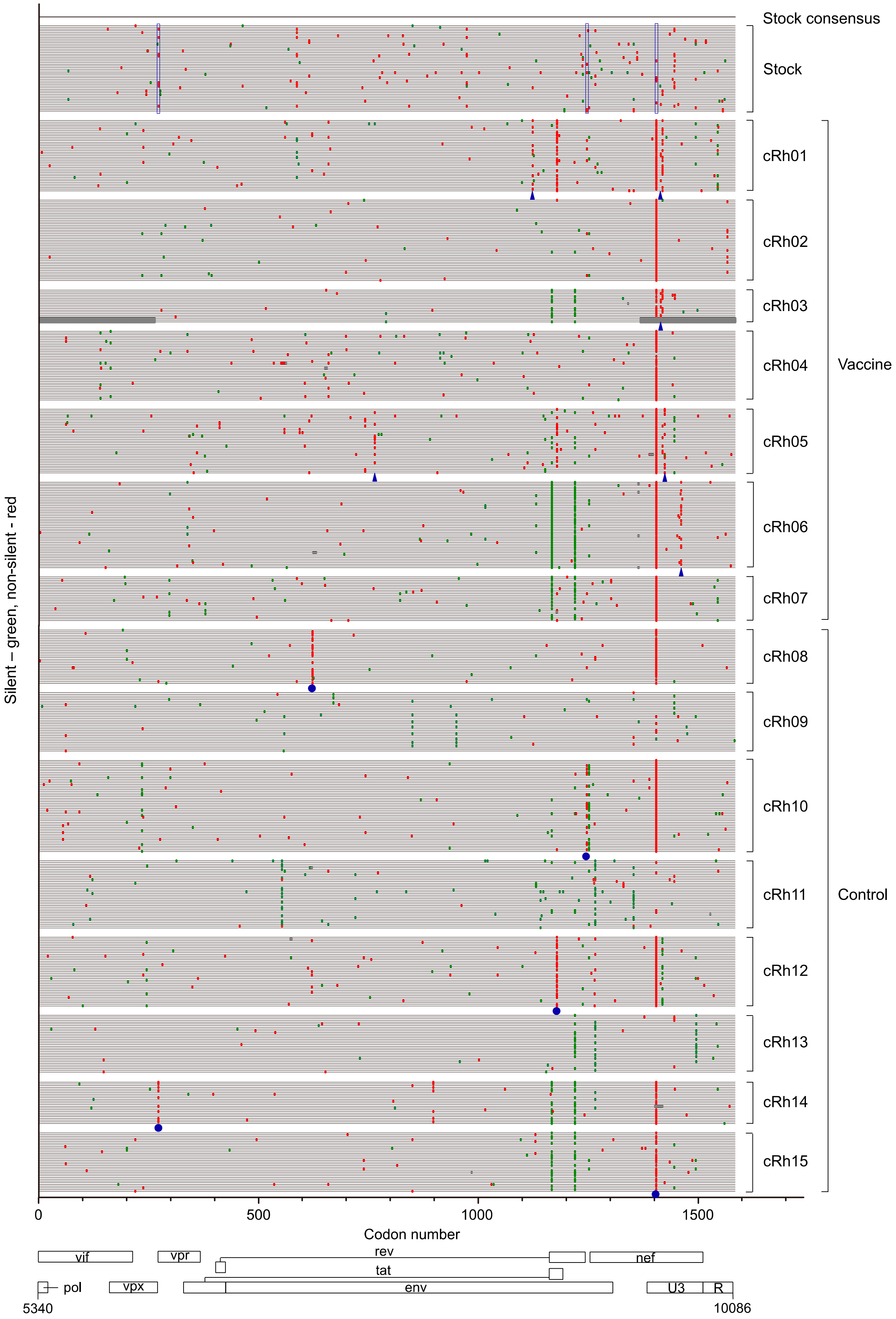

Stock consensus
Stock
cRh01
cRh02
cRh03
cRh04
cRh05
cRh06
cRh07
cRh08
cRh09
cRh10
cRh11
cRh12
cRh13
cRh14
cRh15
Vaccine
Control
Silent – green, non-silent - red
0
500
1000
1500
Codon number
vif
vpr
rev
nef
tat
pol
vpx
env
U3
R
5340
10086

Figure S6. Highlighter plot analysis of 3'-half genome sequences. Multiple 3'-half genome sequences (~4,700 bp) were obtained by SGS for the SIVmac239 challenge stock (n=52) and each plasma sample collected from monkeys 14 days after challenge in both vaccine and control groups (an average of 38; range from 20 to 53). The majority of mutations in the virus population in the challenge stock was random. Sequences from all infected monkeys were compared the challenge virus consensus sequence (top) and the differences were shown by highlighter plot (www.hiv.lanl.gov/content/sequence/HIGHLIGHT/highlighter_top.html). Compared to the SIVmac239 stock consensus sequence, synonymous and non-synonymous mutations detected in viruses from the vaccine and control macaques are indicated by green and red ticks, respectively. Non-synonymous mutations that accounted for 20% or more of the viral population in vaccinated macaques are indicated by blue arrows. Non-synonymous mutations that were selected in control macaques are indicated by blue circles. Mutations that were present in the challenge virus stock and were detected in the unvaccinated macaques are indicated by blue boxes.

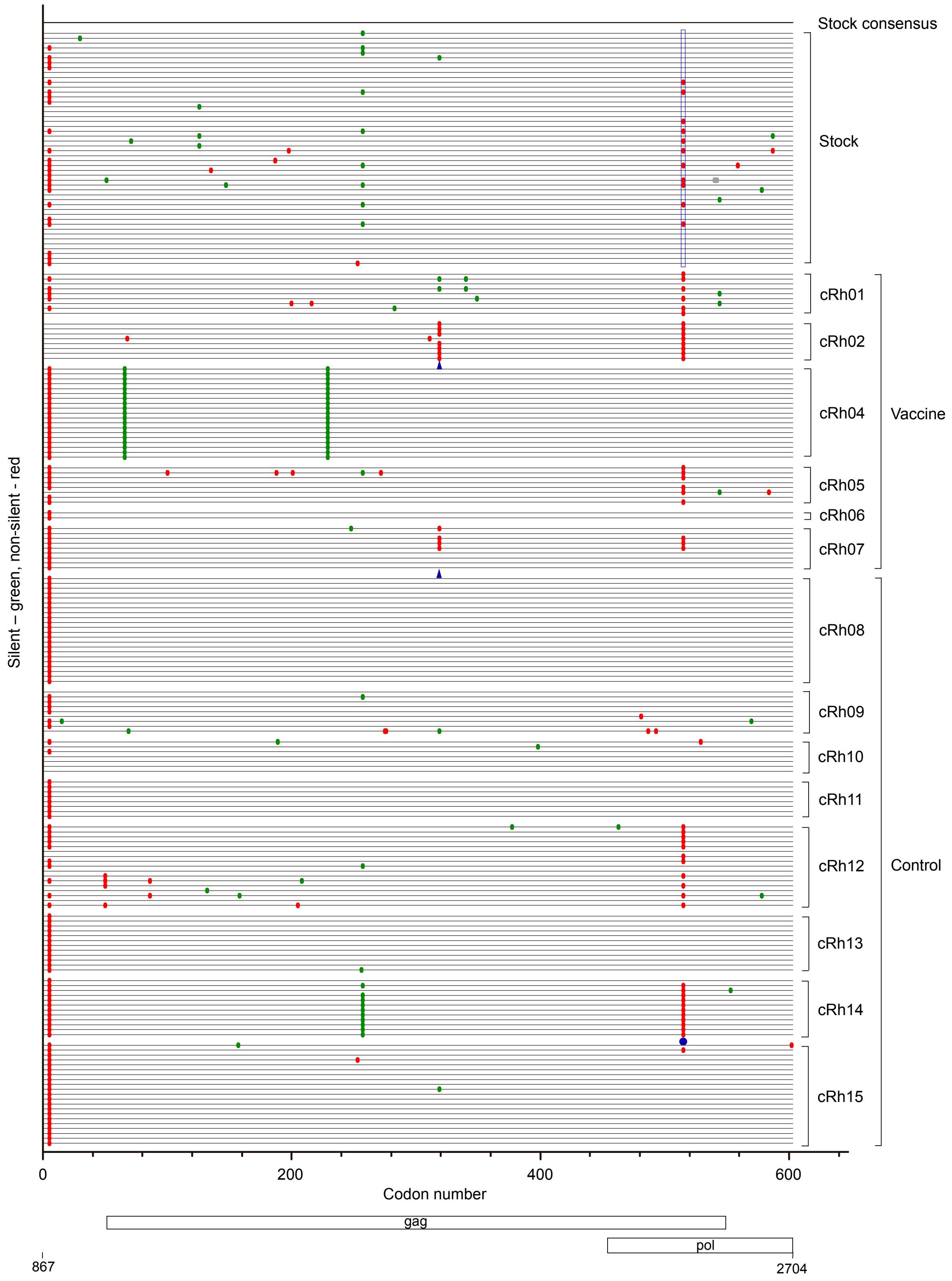
Stock consensus
Stock
cRh01
cRh02
cRh04
cRh05
cRh06
cRh07
Vaccine
cRh08
cRh09
cRh10
cRh11
cRh12
cRh13
cRh14
cRh15
Control
Silent – green, non-silent - red
0
200
400
600
Codon number
gag
pol
867
2704

Figure S7. Highlighter plot analysis of the *gag* gene sequences. Multiple *gag* gene sequences (~1,700 bp) were obtained by SGS for the SIVmac239 challenge stock (n=48) and each plasma sample collected from monkeys 14 days after challenge in both vaccine and control groups (an average of 12; range from 2 to 22). Sequences from all infected monkeys were compared the challenge virus consensus sequence (top) and the differences were shown by highlighter plot (www.hiv.lanl.gov/content/sequence/HIGHLIGHT/highlighter_top.html). Compared to the SIVmac239 stock consensus sequence, synonymous and non-synonymous mutations detected in viruses from the vaccine and control macaques are indicated by green and red ticks, respectively. Non-synonymous mutations that accounted for 20% or more of the viral population in two vaccinated macaques are indicated by blue arrows. Non-synonymous mutations that were detected in both vaccine and control macaques are indicated by blue circles. Mutations that were present in the challenge virus stock and were detected in infected macaques are indicated by blue box.

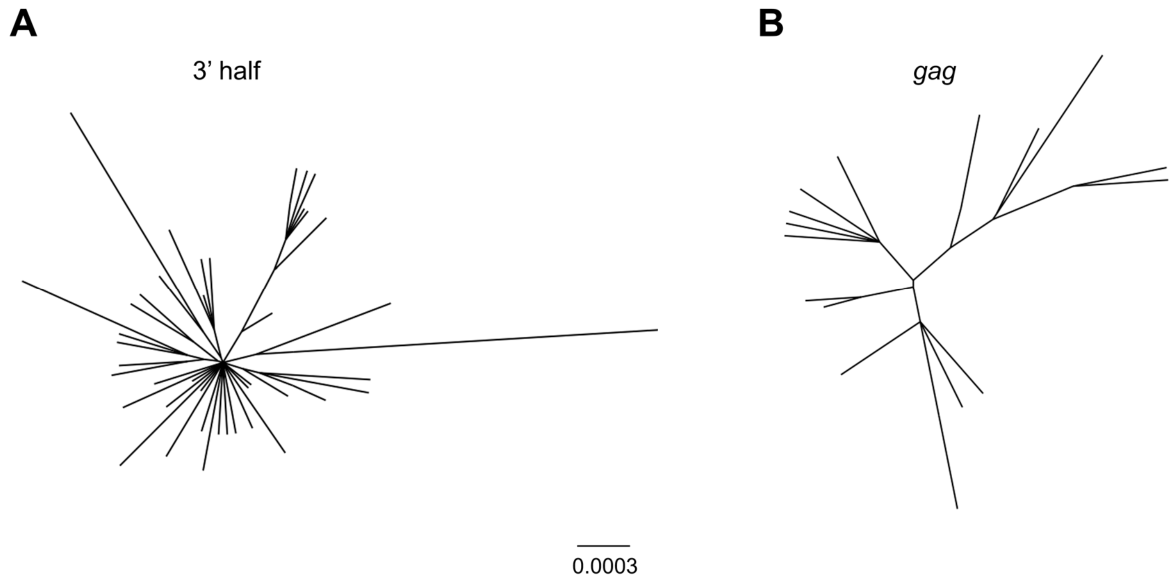

Figure S8. Phylogenetic tree analysis of sequences from the SIVmac239 challenge stock. (A) The 3’-half genome and (**B**) the complete *gag* gene sequences were obtained by SGS from the SIVmac239 challenge stock. Sequences from each region were aligned using CLUSTAL W and the optimal alignments were manually adjusted using Seaview v4. Phylogenetic trees were constructed using the neighbor-joining (NJ) method with Kimura two-parameter mode. The scale bar represents 0.0003 nucleotide substitutions per site.

Table S1. MHC typing of Chinese rhesus macaques

Group	Monkey	MHC alleles			
		*Mamu-A*01*	*Mamu-A*02*	*Mamu-B*08*	*Mamu-B*17*
Vaccine	cRh01	-	-	-	-
	cRh02	+	-	-	-
	cRh03	-	+	-	-
	cRh04	-	-	-	-
	cRh05	-	-	-	-
	cRh06	-	-	-	-
	cRh07	-	-	-	-
Control	cRh08	-	-	-	-
	cRh09	-	-	-	-
	cRh10	-	+	-	-
	cRh11	-	+	-	-
	cRh12	-	+	-	-
	cRh13	-	-	-	-
	cRh14	-	-	-	-
	cRh15	-	-	-	-

"+" means the MHC alleles of an animal is typed as positive, while "-" means negative.

Supplementary Methods

Western-blot analysis. Cells were collected 48 h after transfection and lysed with RIPA (150 mM NaCl, 50 mM Tris, 1% TritonX-100 and 0.1% SDS) buffer. Cell lysates or purified virions were mixed with 4X sample loading buffer (250 mM Tris, 4% β-mercaptoethanol, 40% glycerin, 8% SDS, and 0.4% bromophenol blue) and boiled for 10 min. Viral proteins were separated on a SDS-PAGE, transferred to nitrocellulose membranes, and detected with a SIV$^+$ serum at 1:500 dilution (catalog number 2773, NARP) and the AffiniPure alkaline phosphatase-conjugated goat anti-human IgG secondary Abs (Jackson ImmunoResearch Laboratories, Inc, West Grove, PA) at 1:10,000 dilution. The viral proteins were detected with 0.66% nitro blue tetrazolium (NBT) solution and 0.33% 5-bromo-4-chloro-3-indolyl phosphate (BCIP) solution in 0.1M Tris-HCl (pH 9.5). To detect VSV-G in cotransfected cells and pseudotyped idSIV, VSV-G that was fused with a 9-amino acid marker from hemagglutinin (HA) was detected by an anti-HA MAb at 1:1000 dilution (Covance, Emeryville, CA). The cell lysates were equilibrated by same concentration of tubulin using an anti-tubulin MAb at 1:1000 dilution (Covance) and the ImmunoPure alkaline phosphatase-conjugated goat anti-mouse IgG secondary Abs (Jackson ImmunoResearch Laboratories, Inc) at 1:10,000 dilution.

Detection of viral proteins in infected cells by Immunofluorescence assay. TZM-bl cells were seeded onto coverslips at $5×10^4$ per well in 24-well plates one day before infection. Cell monolayers were about 10 to 20% confluence on the day of infection. After the medium was removed, the same amount of each virus (2.5 ng RT) in 10 ul were mixed with 20 mM DEAE-dextran per well in 200 µl of DMEM, and the mixture was then incubated with TZM-bl cells for 1.5 h at 37°C, followed by addition of 1 ml complete DMEM. After incubation for 48 h, monolayers were washed twice with PBS and fixed for 15 min with 4% paraformaldehyde in PBS. Paraformaldehyde-fixed cells were washed twice with PBS, incubated for 4 min in permeabilizing buffer (0.5% Triton X-100 in PBS), and then blocked using PBS containing 2% bovine serum albumin for 1 h at room temperature. Subsequently, cells were incubated for 1 h at room temperature with an anti-Env mouse monoclonal antibody DA6 (a gift from James Hoxie) or an anti-Nef monoclonal antibody (catalog number 2659, NARP). After three washes, cells were incubated for 30 min with an Alexa 488-lableled anti-mouse antibody or a Rhodamine 570-lableled anti-rabbit antibody (Thermo Fisher Scientific, Waltham, MA). Cellular nuclei were stained with 1 µg/ml of DAPI (49, 69-diamidino-2-phenylindole) in PBS. Images were captured with a confocal laser scanning microscope using a 40X objective (Zeiss, Oberkochen, Germany).

Quantitative detection of integrated and unintegrated proviral genomes. The copy numbers of integrated proviral SIV genomes and unintegrated 2-LTR circles were determined with the second round primers of Alu-PCR and 2-LTR nested-PCR

on an Applied Biosystem 7300 real-time PCR system using a KAPA Library Quantification kit (KAPA Biosystems, Boston, MA, USA). The already-prepared first round products of Alu-PCR and 2-LTR nested-PCR were subjected as the templates for qRT-PCR. The copies of DNA were obtained from the standard curves using the SIVmac239 clone plasmid and the constructed 2-LTR plasmid as standards for the quantitation of integrated proviral SIV genomes and unintegrated 2-LTR circles.

Single genome sequencing. Viral RNA was extracted from SIVmac239 challenge stock and plasma samples collected 14 days post infection using a QIAmp viral RNA kit (Qiagen, Frederick, MD, USA). For 3'-half genome sequences, cDNA was made by reverse transcription using SuperScript III (Invitrogen, Carlsbad, CA) and reverse primer 3R1 (5'-AGGKCTTTAAGCAAGCAAGCGTGGA-3'; nt 10103–10127). SGS of cDNA was carried out with cDNA samples. PCR products from dilutions that yielded ~20% positive amplification were subjected to sequencing analysis. First-round PCR was performed with 0.2 μM of forward primer 3F2-2S (5'-GGACCCGGTGARCTAYTGTGGAAAGGGGAAG-3'; nt 5237–5267) and reverse primer 3R2 (5'-AAGCGTGGAGYCACTCTGCCCAGCACCGGCCAAG-3'; nt 10078–10111), and 0.5 U High Fidelity Platinum *Taq* DNA Polymerase (Invitrogen) in a 20 μl reaction containing High fidelity buffer, 2 mM $MgSO_4$ and 0.2 mM of dNTP. PCR conditions were 94°C for 2 min, followed by 35 cycles of 94°C for 15 s, 64°C for 30 s, and 68°C for 5 min, with a final extension period of 10 min at 68°C. Second-round PCR was performed with 0.2 μM of forward primer 3F3 (5'-GCTAAAATTATCAAAGAYTATGSAGGAGGAAAAGA-3'; nt 5321–5355) and reverse primer 3R3 (5'-CCGGCCAAGTGCTGGTGAGA-3'; nt 10067–10086), 1.25 U High Fidelity Platinum *Taq* DNA Polymerase and 2 μl of the first-round product in a 50 μl reaction. The PCR conditions were the same as for the first-round PCR, except that the annealing temperature was decreased to 60°C.

For the *gag* gene SGS analysis, cDNA was made by reverse primer 5R3 (5'-CCGCTGWAAAGCAAGNGAAATAAGT-3'; nt 5673–5697). The first-round primers were 5F1 (5'-CTGAACAGGGACTTGAAGGAGAG-3'; nt 828–850) and 5R1 (5'-GGCGACTTTTACAGGCTCTACTT-3'; nt 2862–2884). The second-round primers were (5'-ACGGCTGAGTGAAGGCAGTAAGG-3'; nt 867–889) and (5'-TATTCCTCCTCTATTTTTGGGG-3'; nt 2682–2704). The PCR conditions were the same as for the 3' half PCR, except that the annealing temperature was 55°C for both of the first- and the second-round PCR.

PCR products were purified using a High Pure PCR Product Purification Kit (Roche, South San Francisco, CA). Sequencing library for each amplicon was prepared using the Nextera XT Library Prep Kit (Illumina, San Diego, CA). Each library was indexed using the index primers from a Nextera XT Index Kit (Illumina). Up to 96 libraries were polled together and sequenced using MiSeq (Illumina).

Sequence analysis. Raw sequence reads obtained by high-throughput sequencing were assembled and the consensus sequence for each PCR amplicon without mixed bases at any position were generated using Geneious v9.0.4. The final sequences were aligned together with the challenge SIVmac239 stock virus sequence using CLUSTAL W and manual adjustment for optimal alignment was done using Seaview v4. Phylogenetic trees were constructed using the neighbor-joining (NJ) method with Kimura two-parameter model, and the reliability of topologies was estimated by bootstrap analysis with 1000 replicates. Highlighter analysis was performed through the LANL database website (http://www.hiv.lanl.gov/content/sequence/HIGHLIGHT/highlighter.html).

References

1. Guan, Y.; Whitney, J.B.; Detorio, M.; Wainberg, M.A. Construction and in vitro properties of a series of attenuated simian immunodeficiency viruses with all accessory genes deleted. *J Virol* **2001**, *75*, 4056-4067.
2. Sloan, R.D.; Wainberg, M.A. The role of unintegrated DNA in HIV infection. *Retrovirology* **2011**, *8*, 52.
